# Knowledge translation in health: how implementation science could contribute more

**DOI:** 10.1186/s12916-019-1322-9

**Published:** 2019-05-07

**Authors:** Michel Wensing, Richard Grol

**Affiliations:** 1 Department of General Practice and Health Services Research, Heidelberg University Hospital, Im Neuenheimer Feld, 130.3, 69120 Heidelberg, Germany; 2 Radboud Institute for Health Sciences, Radboud University Medical Center, Geert Grooteplein Zuid 10, 6525 Nijmegen, GA Netherlands; 3 Maastricht University, Minderbroedersberg 4-6, 6211 Maastricht, LK Netherlands

**Keywords:** Knowledge transfer, Implementation science, Quality improvement, Research policy

## Abstract

**Background:**

Despite increasing interest in research on how to translate knowledge into practice and improve healthcare, the accumulation of scientific knowledge in this field is slow. Few substantial new insights have become available in the last decade.

**Main body:**

Various problems hinder development in this field. There is a frequent misfit between problems and approaches to implementation, resulting in the use of implementation strategies that do not match with the targeted problems. The proliferation of concepts, theories and frameworks for knowledge transfer – many of which are untested – has not advanced the field. Stakeholder involvement is regarded as crucial for successful knowledge implementation, but many approaches are poorly specified and unvalidated. Despite the apparent decreased appreciation of rigorous designs for effect evaluation, such as randomized trials, these should remain within the portfolio of implementation research. Outcome measures for knowledge implementation tend to be crude, but it is important to integrate patient preferences and the increased precision of knowledge.

**Conclusions:**

We suggest that the research enterprise be redesigned in several ways to address these problems and enhance scientific progress in the interests of patients and populations. It is crucially important to establish substantial programmes of research on implementation and improvement in healthcare, and better recognize the societal and practical benefits of research.

## Background

Across the world, decision makers in healthcare struggle with the uptake of rapidly evolving scientific knowledge into healthcare practice, organisation, and policy. Rapid uptake of high-value clinical procedures, technologies, and organisational models is needed to achieve the best possible healthcare outcomes. Perhaps an even bigger struggle is that of stopping practices that do not or no longer have high value, such as the use of antibiotics for mild respiratory infections. Targeted interventions to improve healthcare practice exist in nearly all countries, and include, for instance, financial incentive programs to enhance the performance of healthcare providers, continuing professional education, and tools to involve patients more actively in their care and enhance shared decision-making. Evaluations of such implementation interventions in realistic settings found mixed, and overall moderate effects [1, 2]. As a consequence, there have been calls to harness research and development on this topic [3]. We believe, however, that in recent years there has been little progress in our understanding of how healthcare practice can be improved.

The growing field of research on how to improve healthcare is known under various names, such as quality improvement, (dissemination and) implementation research, and knowledge transfer or translation [4]. *Implementation science* has been defined as the “scientific study of methods to promote the systematic uptake of research findings and other evidence-based practices into routine practice, and, hence, to improve the quality and effectiveness of health services and care” [3]. *Knowledge translation* is a related field that aims to enhance the use and usefulness of research. It covers the design and conduct of studies, as well as the dissemination and implementation of findings [4]. In healthcare, *quality and safety management* comprises a set of activities that aim to improve healthcare quality and safety, using measurement, feedback to decision-makers and organizational change [5]. In this article, we consider these and related fields as largely overlapping, because, through better uptake of putting knowledge in practice, they all aim to improve healthcare practice and thus provide better outcomes for patients and populations [6]. Knowledge may take various forms, such as evidence-based practice guidelines, technologies, or healthcare delivery models with proven value. The field is usually associated with change of practice and behaviors, but resisting change may occasionally serve the same purpose (e.g. in the case of promoted changes for which the evidence is limited or negative).

An illustrative example of research on knowledge transfer in health concerns pay-for-performance in general practice, which was introduced more than a decade ago in the UK. Practice performance was operationalized in terms of performance indicators, for which many had strong underlying evidence. Higher scores were associated with higher financial payments. For instance, an interrupted time series analysis of changes in performance scores for diabetes, asthma, and coronary heart diseases between 1998 and 2007 showed substantial improvements. These improvements had started before 2004; the year in which the contract was introduced. In diabetes and asthma, but not for coronary heart disease, a significant acceleration of improvement has been found since 2004 [7]. Other research in the field has focused, for instance, on continuing education, organizational change, health system reforms, and patient involvement for improving healthcare and implementation of recommended practice.

Looking at the infrastructure for research on how to improve healthcare practice, many positive developments can be noted in recent decades. Major funders, such as the National Institute for Health Research in the UK, the National Institutes of Health in the USA, the Canadian Institutes of Health Research, and Innovationsfond in Germany, have made substantial funding available. Several large research programs (funded with many millions of euros, pounds or dollars) have been established, such as the Collaborations for Leadership in Applied Health Research and Care (CLAHRCs; soon to be ARCs) in the UK. Scientific journals dedicated to the field have emerged, such as *BMJ Quality & Safety*, and *Implementation Science*, and many medical and health science journals publish research on healthcare improvement. Professorships and training programs for implementation science and quality improvement have been created, most notably in North America, but also in other countries. A variety of yearly or biannual scientific conferences focus on healthcare improvement, implementation science, and related fields.

Despite this emerging infrastructure and growing interest in the field, it seems to lag behind the high expectations. We are still far from understanding how healthcare practice can be improved rapidly, comprehensively, at large scale, and sustainably. In fact, this observation has been made several times in previous decades. For instance, in the year 2000, reflecting on 20 years of implementation research in healthcare, Grol and Jones argued that many questions had remained unanswered [8]. Seven years later, Grol, Wensing and Berwick argued that research on quality and safety in healthcare must be strengthened to develop the science in this field [9]. In 2014, Ivers and colleagues suggested that science is stagnating, based on an analysis of audit and feedback strategies [10]. We feel that progress in the field has been limited in the previous decade. This Opinion article focuses on what may be wrong in research and practice, and what could help to address the problems. It is informed by our research projects, lectures, teaching at graduate and postgraduate levels, participation in academic review committees, roles as journal editors, and repeated updates of our books on improving healthcare practice [11, 12] in the previous 25 years.

### Problems and possible strategies

Table 1 provides an overview of our analysis of problems in research and practice, and possible ways to address these.

**Table 1:** Challenges in research on how to improve healthcare practice

Issue	Current practice	Desired practice	Possible strategies
Fit between problems and approaches to address these	Approach depends on the professional background of the practitioner, researcher or advisor	Approach depends on what fits best with the problem in healthcare practice	Researchers and advisors are trained in a variety of scientific disciplines and work in multidisciplinary teams
Concepts, frameworks and theories for implementation, transfer and improvement	Proliferation of proposals; many are descriptive lists of items; testing and validation strictly within paradigms	Emphasis on testing, integration and refinement of middle-range theories	Better recognition of published work; funding of comparison across paradigms
Stakeholder involvement in design and conduct of programs	Considered to be the single most important determinant of successful implementation, but most methods are loosely defined	Integration of evidence and theory with of stakeholder involvement, using well-specified approaches	Validation of structured approaches for stakeholder involvement
Evaluation of outcomes of implementation programs	Decreased interest in rigorous effect evaluations of implementation interventions, such as randomized trials	Rigorous outcomes evaluation remain part of broader research programs, which likely comprise a variety of study designs and methods	Nurture knowledge and appreciation of rigorous evaluation of practices among healthcare practitioners, managers and policy makers
Measurement of outcomes of improvement, transfer, and implementation	Descriptive documentation of professional practice, provider perceptions, or health outcomes	Advanced measures of rapid uptake of valuable practices, and rapid reduction of non-valuable practices	Design and validation of a new generation of outcome measures

#### Misfit between problems and approaches

Healthcare challenges vary widely. For instance, implementing the centralization of surgical procedures or emergency services in specific centers is very different from a change in a medication protocol, or the introduction of nurses for counseling on lifestyle changes. All of these changes may be backed up by strong research evidence on their value, but analysis of the implementation challenges in each of these examples would reveal very different determinants of implementation and outcomes. For instance, health system factors may be crucial for the effective centralization of services, while individual factors related to knowledge or behavioral routines are probably most relevant in changing medication protocols. However, there is often little association between the type of problem and the approach to change taken. More particularly, organizational and system-related problems tend to be ignored, even when these were detected, favoring individual educational and psychological approaches [13]. Organizational and system change may be difficult to achieve within the typical course of a research project.

Probably even more relevant is that many researchers and consultants tend to be stubbornly consistent in their approach, and stick, for instance, either to a psychological approach (arguing that all implementation requires individual behavior change), an organizational systems approach (arguing that organizations rather than individuals need to change), or an economic perspective (arguing that financial factors override everything else). We believe, however, that the chosen approach to change should match the problems to achieve change, rather than the favored discipline of the researcher or consultant [14]. To remedy the current situation, options include training researchers and consultants and providing them with a multi-faceted perspective on the issue of knowledge translation and with theoretical frameworks that cover multiple approaches, as well as working in multidisciplinary improvement teams. These approaches require multidisciplinary academic training to be better appreciated in universities, which tend to facilitate academic careers within traditional scientific disciplines.

#### Proliferation of concepts, theories and frameworks

In the past [15], research on the translation of knowledge into practice was dominated by a few theories, such as the ‘Diffusion of Innovations’ theory [16]. Nowadays, research on improving healthcare practice is characterized by a proliferation of concepts, theories and frameworks for knowledge translation and implementation [17]. Most are essentially structured lists of disconnected items, which are not explicitly linked to higher-level scientific theory [18]. They distinguish, for instance, between individual, organizational, system-related and innovation-related factors. Many of these proposed frameworks have not been applied and tested in more than one study. New frameworks typically ignore published work – particularly if it is older than a decade – so the reinvention of existing concepts and frameworks is common. The field has also suffered from many fashions and hypes, which often present high-level concepts (e.g. the ‘breakthrough’ approach to improvement) and were popular for a while, but then disappeared without contributing much to scientific progress.

Research that applies and tests concepts, theories or frameworks is largely organized in silos – bubbles of like-minded academics (e.g. epidemiologists, psychologists, sociologists, or economists). Among researchers, there is mixed interest in testing conceptual ideas in rigorous empirical research. Some are close to healthcare practice (e.g., as clinicians), but are not aware of the full range of available concepts, theories and frameworks. Others know and apply specific theories or frameworks, but may be insufficiently familiar with healthcare practice or policy to assess their usefulness. Furthermore, head-to-head comparisons and integrations of proposals from different silos are rare, because many researchers are inclined to stick to their favored approach. We suggest, however, that these would advance science far more than the continuous development of new theories within disconnected worlds. Rather than adding new frameworks, the focus should be on the testing, refinement and integration of theories. Furthermore, we think that these should be sufficiently concrete and specific. To be sufficiently informative, they may be related to a specific topic or field of application – although it remains open for debate as to what would be the appropriate aggregation level (e.g., antibiotics prescribing in primary care, medication prescribing generally, or ambulatory medical care).

#### Non-validated methods for stakeholder involvement

The involvement of stakeholders (e.g., patients, providers, payers) in the design and conduct of interventions to improve healthcare practice has been emphasized to the extent that it is now seen as the ‘holy grail’ of improvement. For instance, ‘mode 2’ knowledge generation has been characterized as socially distributed, organizationally diverse, application-oriented, and transdisciplinary, as opposed to the traditional, ‘ivory tower’ mode 1 [19]. Involvement usually takes the form of consultation of stakeholders through interviews or surveys, or the participation of stakeholders in boards. However, the evidence for this belief is – so far – largely anecdotal, and many methods for stakeholder involvement are poorly specified, so replication is difficult. For instance, integrated knowledge translation (collaboration between researchers and decision-makers) uses a variety of methods, but its outcomes are unknown [20]. Stakeholders are often heterogeneous with respect to knowledge, needs and preferences regarding a particular change. Attempts to develop and validate stakeholder-based, tailored interventions have met with various difficulties and uncertainties [21]. For example, it is unclear how available research evidence and theory is combined with stakeholder involvement, if stakeholders have suggestions that contradict existing knowledge. Stakeholder involvement also implies the use of resources, particularly health professionals’ time, which must be considered when planning implementation programs. We suggest that methods for stakeholder involvement must be better specified and validated in empirical research.

#### Decreased appreciation of rigorous designs for effect evaluation

In some circles of researchers, practitioners, and policy makers, there seems to have emerged a decreased appreciation of rigorous designs for effect evaluation in healthcare, such as randomized trials. Arguments for the criticism of randomized trials as a preferred study design are manifold, and include, for instance, the belief that many interventions are changed during application in practice, outcomes of interventions are largely context-dependent, rigorous evaluation is time-consuming and expensive, and biomedical knowledge is evolving too quickly to allow rigorous outcomes evaluation [22]. Implementation science needs a variety of study designs and methods, including systematic intervention development, observational pilot tests, qualitative studies, and quantitative simulation modeling. We believe that rigorous designs for effect evaluation, including randomized trials, should remain on the menu.

Several researchers appreciate randomized trials and other rigorous evaluation designs, but they focus on the effectiveness of interventions with respect to patients’ health. Implementation researchers can indeed make useful contributions to process evaluation and feasibility testing of interventions in studies of intervention effectiveness. While such research is important, it is unlikely to advance implementation science much, because this is not its primary objective, and, as a consequence, the methods are not fully aligned. For instance, clinical trials and trials in public health often benefit from optimized intervention fidelity and strict inclusion criteria for participants, which does not match with the requirements of research on quality improvement, knowledge translation and implementation science.

There are clearly various issues that must be carefully considered when designing rigorous evaluations of improvement and implementation interventions, such as the choice of outcome measures, the duration of follow-up, and the approach in control groups. Several trial designs, such as stepped wedge trials, provide options beyond the classic two-armed randomized trial – albeit often at the price of more complex statistical analysis. Advanced designs for rigorous evaluation can only be considered if the people involved understand and appreciate outcome evaluation in the first place. It cannot be assumed that this is the case. We therefore argue that the appreciation of outcome evaluation must be nurtured among healthcare providers, managers and policy-makers. This appreciation should extend to interventions for the improvement of healthcare practice.

#### Suboptimal outcomes measures

Strategies for improvement or implementation must be evaluated with respect to their effectiveness in changing healthcare practices as a primary outcome of interest. Many studies of interventions to improve healthcare practice use relatively simple outcomes measures, such as clinical behaviors, which have been documented in patient records or procedures for which reimbursement was requested. While such measures can be informative, they are often only crude indicators of the actual use of knowledge in healthcare practice. Recent and continuing work on the pragmatic use of outcomes in implementation research has emphasized the importance of acceptability, feasibility, compatibility with routines, and perceptions of usefulness [23]. In some studies, however, health outcomes are primary outcomes – although these may not be responsive for improvements in the quality of care. The number of steps from interventions to health outcomes is relatively great, so that interpretation of causality is difficult if intermediate factors are not measured – particularly if health outcomes do not show intervention effects.

Furthermore, a fundamental problem is that the use of knowledge in decision-making rarely has simple, predictable associations with the decisions made. Ultimately, the key outcome is not crude frequencies of behaviors or health outcomes, but whether the available knowledge was taken into consideration in decision-making and healthcare practice. This knowledge is increasingly individualized on the basis of patients’ biological and psychological features, and comes from computerized decision support systems rather than clinical guidelines for patient populations. Patients’ preferences must also be taken into account when assessing the quality of decision-making. A knowledge-informed conversation between a patient and a clinician, resulting in a decision that is not coherent with some recommendation, should not automatically be documented as non-use of knowledge. We believe there is a need for new outcome measures of knowledge implementation, which take the changing nature of knowledge and the impact of patient preferences into account.

## Discussion

A fundamental challenge is to overcome the misconceptions, silo-thinking and self-interests among stakeholders. These stakeholders include politicians and managers who prefer to act on the basis of conviction rather than research evidence, healthcare providers who deny that research findings apply to them, and researchers who prefer to focus on concepts and approaches that fit their particular academic background. Global investment in the research infrastructure of knowledge implementation and quality improvement in healthcare provides major opportunities, but also a high responsibility for all involved.

After participating in many grant review panels, we conclude that the assessment of project applications is often dominated by the perceived relevance of the health issue or healthcare problem on which it focuses. The project’s contribution to the agenda of research on how to improve healthcare practice is usually of secondary importance, at best. This practice of healthcare research funding has resulted in many researchers in the field who do one or two projects on how to improve healthcare practice, and then leave the field, because they see no perspective for a career, or lack real interest. As a result, research on healthcare improvement and knowledge implementation is currently fragmented in different, largely disconnected, communities [24].

We think that coordinated and longer lasting research programs are needed to enhance the continuity of researchers in the field, which is crucial for knowledge accumulation. Knowledge translation and healthcare improvement in healthcare needs research centers or networks that bring together scientists with different backgrounds who can work on sequential projects over a longer period of time. Examples exist, and include an international research network to examine and optimize feedback interventions for implementation [25], and a large center for the study of healthcare improvement [26]. Effective programmatic research requires, among other things, institutional funding for core staff, multidisciplinary composition of groups, realistic and continuous funding opportunities for research projects, career opportunities for young and mid-career researchers, and integration in locally relevant infrastructures (e.g., routine quality improvement in hospitals). It also requires focused education. For instance, some graduation programs for implementation science in healthcare have been established [24], but their long-term success depends on graduated students’ opportunities in the labor market.

The field would also be enhanced by the revision of procedures for accountability and recognition of performance in academic institutions. Research on healthcare improvement and implementation is unlikely to provide ‘discoveries’, but studies can add substantially to the body of knowledge and thus support users in practice, management and policy. Citations in scientific journals are problematic as a sole criterion for review of performance because users (e.g. clinicians, managers, policy-makers) rarely cite publications, as most do not publish scientific papers. Several complementary methods for performance review are available, but their validity and feasibility remain challenging [27]. Perhaps most importantly, research on improving healthcare and knowledge implementation requires a higher appreciation of the field in the academic and health community, and alignment of resources and power in institutions accordingly.

## Conclusions

The ultimate aim of research on knowledge implementation in healthcare is for interventions to improve healthcare practice to become more effective, thus leading to better care and outcomes for patients and populations. We believe that the research enterprise in this field must be redesigned in several ways to make this happen.
